# MSATNet: multi-scale adaptive transformer network for motor imagery classification

**DOI:** 10.3389/fnins.2023.1173778

**Published:** 2023-06-14

**Authors:** Lingyan Hu, Weijie Hong, Lingyu Liu

**Affiliations:** ^1^School of Information and Engineering, Nanchang University, Nanchang, Jiangxi, China; ^2^School of Electronic and Electrical Engineering, Shanghai University of Engineering Science, Shanghai, China; ^3^School of Qianhu, Nanchang University, Nanchang, Jiangxi, China; ^4^Shanghai Yangzhi Rehabilitation Hospital (Shanghai Sunshine Rehabilitation Center), Shanghai, China

**Keywords:** electroencephalogram, motor imagery classification, multi-scale convolution, transformer, transfer learning

## Abstract

Motor imagery brain-computer interface (MI-BCI) can parse user motor imagery to achieve wheelchair control or motion control for smart prostheses. However, problems of poor feature extraction and low cross-subject performance exist in the model for motor imagery classification tasks. To address these problems, we propose a multi-scale adaptive transformer network (MSATNet) for motor imagery classification. Therein, we design a multi-scale feature extraction (MSFE) module to extract multi-band highly-discriminative features. Through the adaptive temporal transformer (ATT) module, the temporal decoder and multi-head attention unit are used to adaptively extract temporal dependencies. Efficient transfer learning is achieved by fine-tuning target subject data through the subject adapter (SA) module. Within-subject and cross-subject experiments are performed to evaluate the classification performance of the model on the BCI Competition IV 2a and 2b datasets. The MSATNet outperforms benchmark models in classification performance, reaching 81.75 and 89.34% accuracies for the within-subject experiments and 81.33 and 86.23% accuracies for the cross-subject experiments. The experimental results demonstrate that the proposed method can help build a more accurate MI-BCI system.

## 1. Introduction

A brain-computer interface (BCI) establishes a direct connection between the human brain and a computer or external device, without requiring muscular stimulation (Saha et al., 2021). A BCI system decodes the patient’s intentions to move specific limbs, and subsequently uses these decoded intentions to provide corresponding sensorimotor feedback to the patient in various forms (Mane et al., 2020). In addition to being widely employed in the field of medical rehabilitation, BCI offers significant room for growth in fields like sleep monitoring, brain disease detection, and game entertainment (Chen et al., 2018; Arpaia et al., 2020; Lee et al., 2021).

As a non-invasive approach, motor imagery brain-computer interface (MI-BCI) has the characteristics of high safety and low power consumption. Motor imagery can alter neuronal activity in primary sensorimotor areas in a manner similar to that of performing actual movements (Pfurtscheller and Neuper, 2001). When motor imagery occurs, energy in different sensory regions of the cerebral cortex changes and leads to event-related desynchronization (ERD) and event-related synchronization (ERS). Compared to other BCI paradigms, motor imagery is stimulus-independent and does not require external stimuli (Khan et al., 2020). A multi-scale adaptive transformer network (MSATNet) is designed in this paper to classify and recognize users’ motor imagery intention by acquiring their electroencephalogram (EEG) signals through MI-BCI. The category information predicted by the network can be used as output commands for wheelchair control or motion control of smart prostheses.

Due to the low signal-to-noise ratio and non-stationarity of EEG signals, it is difficult to extract features with high discrimination abilities. Therein, traditional machine learning methods have been widely applied to EEG decoding. The main steps include feature extraction and classification. For feature extraction, EEG features are divided into temporal features, spectral features, and spatial features. Temporal features are extracted from time points or time segments, such as by the mean and variance, while spectral features include frequency-domain and time-frequency features, such as the power spectral density and wavelet transform. The common spatial pattern (CSP) (Ramoser et al., 2000) is the most widely-used spatial feature extraction algorithm, and many researchers have attempted to improve its baseline implementation. To mitigate the negative impact of outliers and noise on the performance of conventional CSP method, researchers have proposed several modifications and enhancements, such as Sparse Common Spatial Pattern (SCSP)(Arvaneh et al., 2011), CSP-L1(Wang et al., 2012), CSP-QMEE (Chen et al., 2020). These modifications can enhance the robustness and accuracy of the CSP algorithm and have shown promising results in improving the performance of BCIs. For the classification stage, several classifiers have been used to distinguish high-dimensional features, such as the support vector machine (SVM) and linear discriminant analysis (LDA). However, these methods rely on feature selection and require extensive professional experience.

Deep learning can automatically perform representation learning without tedious preprocessing and feature engineering. Two classical deep learning models are the convolutional neural network (CNN) and recurrent neural network (RNN), which are widely used for EEG classification in motor imagery. Existing CNN models can be divided into two types based on the different input forms of the model. One is to directly input the original signal into the model, such as deep Convnet (Schirrmeister et al., 2017) and EEGNet (Lawhern et al., 2018), and the other is to input the extracted features into the model. Xue et al. used the FBCSP algorithm to extract spatial features and the multilayer brain network into their respective CNN models before finally performing feature fusion (Xue et al., 2020). Sujit Roy et al. used the short-time Fourier transform to extract time-frequency maps of the EEG as the model input (Roy et al., 2020).

Existing CNN-based methods perform better in terms of classification performance, but most only use single-scale convolution, which is inadequate to extract EEG signals using individual and temporal variability and has poor recognition accuracy. Dai et al. improved the accuracy of EEG classification using mixed-size convolution kernels for feature extraction (Dai et al., 2020). Inspired by the inception network structure in computer vision, EEG-Inception uses one-dimensional convolutions of different sizes for feature extraction (Zhang et al., 2021). Further, Jia et al. used a multi-branch multi-scale structure to achieve state of the art (SOTA) in EEG recognition (Jia et al., 2021). However, these methods have too many model training parameters and are prone to overfitting in the face of small datasets, which limits their recognition performance.

Recent studies have found the existence of long-range temporal correlation (LRTC) in EEG signals during motion imagery, which changes dramatically over time (Wairagkar et al., 2021). Therefore, capturing long-range temporal dependencies in EEG signals is important for feature extraction. To this end, many studies have applied RNN-based models for temporal modeling. Wang et al. used a one-dimensional aggregation approximation for dimensionality reduction and input the results into the long-short term memory (LSTM) for feature extraction (Wang et al., 2018). The gate recurrent unit (GRU) simplifies the model structure and improves the training efficiency. Liu et al. applied the GRU to extract the temporal dependence in deep features (Liu et al., 2022). Although these methods exploit the time series features of EEGs, the models are too complex for parallel training. The temporal convolutional network (TCN) improves upon these drawbacks, and the training efficiency is greatly improved as no gradient disappearance or gradient explosion occurs when training on long input sequences (Ingolfsson et al., 2020). However, the above methods can only mine a small range of time-dependent relationships, and there are still limitations when modeling long-sequence EEG signals.

An important function of a practical MI-BCI system is to accurately recognize different subjects. Although previous models have achieved high performances for within-subject tests, these models are heavily data-dependent due to the individual variability of EEG data, which results in poor model generalization performance. To address these issues, transfer learning, which is a machine learning method that reduces data shifts between different domains, has achieved better performance in BCI classification. Domain adaptation is a common transfer learning approach that reduces the gap between the source and target domains *via* feature transformation. Wei et al. (2021) proposed aligning feature distributions using the maximum mean discrepancy. Chen et al. then used domain adversarial training to reduce the gap in the depth features between individuals (Chen et al., 2022). However, this domain adaptation-based approach requires access to the data of all individuals in the target domain, which is difficult to implement in practice.

To solve these problems, we propose a novel multi-scale adaptive transformer model (MSATNet) for motor imagery decoding to obtain user motion imagery intention from collected EEG data. The contributions of this paper are given as follows.

1. In terms of feature extraction, in order to solve the problem that the models in the past methods are too complex and prone to overfitting, we propose a Multi-Scale Feature Extraction (MSFE) Module, which uses two branches and different convolution kernels to extract features of different frequency bands.
2. To address the limitation of previous methods, which could only capture small-scale time dependencies, we propose an Adaptive Temporal Transformer (ATT) Module. By combining temporal convolution with multi-head attention mechanism, we were able to capture long-range temporal dependencies from deep features of EEG signals.
3. In terms of model generalization, in order to solve the limitations of previous methods that need to obtain all the data of the target subject, we designed a Subject Adapter Module. By fine-tuning a pre-trained model with a small amount of data from new individuals, we attained good performance.

The rest of the paper is organized as follows. Section 2 describes the structure of MSATNet, Section 3 describes the experimental setup, Section 4 analyzes the experimental results, and conclusions are drawn in Section 5.

## 2. Materials and methods

### 2.1. Data description

This paper uses the BCI Competition IV 2a and 2b datasets (Tangermann et al., 2012) to evaluate the validity of the proposed model. The BCI Competition IV 2a is a multiclass motor imagery dataset that contains EEG recordings from nine participants during imagining movements for the left hand, right hand, feet, and tongue. Data were collected through a band pass filter from 0.5–100 Hz with a sampling rate of 250 Hz. The signals consist of 22 EEG channels and 3 electro-oculogram (EOG) channels. Based on the requirements of the dataset, we removed the data of the EOG channel during the experiments. In addition, the dataset consists of two sessions that were collected on different days for each subject. Each session consists of 288 trials with the same number of trials for each category. The first session was used as the training set, while the second session was used as the test set. Each trial in the paradigm began with a 2 s prep time and was followed by a cue that lasted 1.25 s to represent the imagined class. The imagination period lasted 4 s after the cue was initiated and was terminated by the rest period.

The BCI Competition IV 2b is an EEG dataset based on visually evoked left- and right-handed motor imagery. The EEG signals of nine participants were collected in the dataset. Each experimenter’s EEG data set consists of five sessions, the first two sessions are EEG imagery data without visual feedback, and the last three sessions are EEG imagery data with visual feedback. Each session with visual feedback had a total of 120 motor imagery data segments, and each session without visual feedback contained 160 motor imagery EEG segments. We utilized a total of five sessions from the dataset, with the first three sessions used as the training set and the remaining two sessions used as the test set. The data in all experiments were band-pass filtered at 0.5–100 Hz and trap filtered at 50 Hz. The sampling frequency of the entire experiment was 250 Hz.

### 2.2. EEG representations

The initial EEG signal is defined as $\left\{\left(X_{i},y_{i}\right)|i=1,2,\ldots ,K\right\}$ , where $X_{i}\in R^{C\times T}$ is a representation of the i -th trial consisting of C channels and T sampling time points, $y_{i}$ is the sample label corresponding to $X_{i}$ , and K is the total number of trials.

### 2.3. Overall model framework

**Figure 1:**
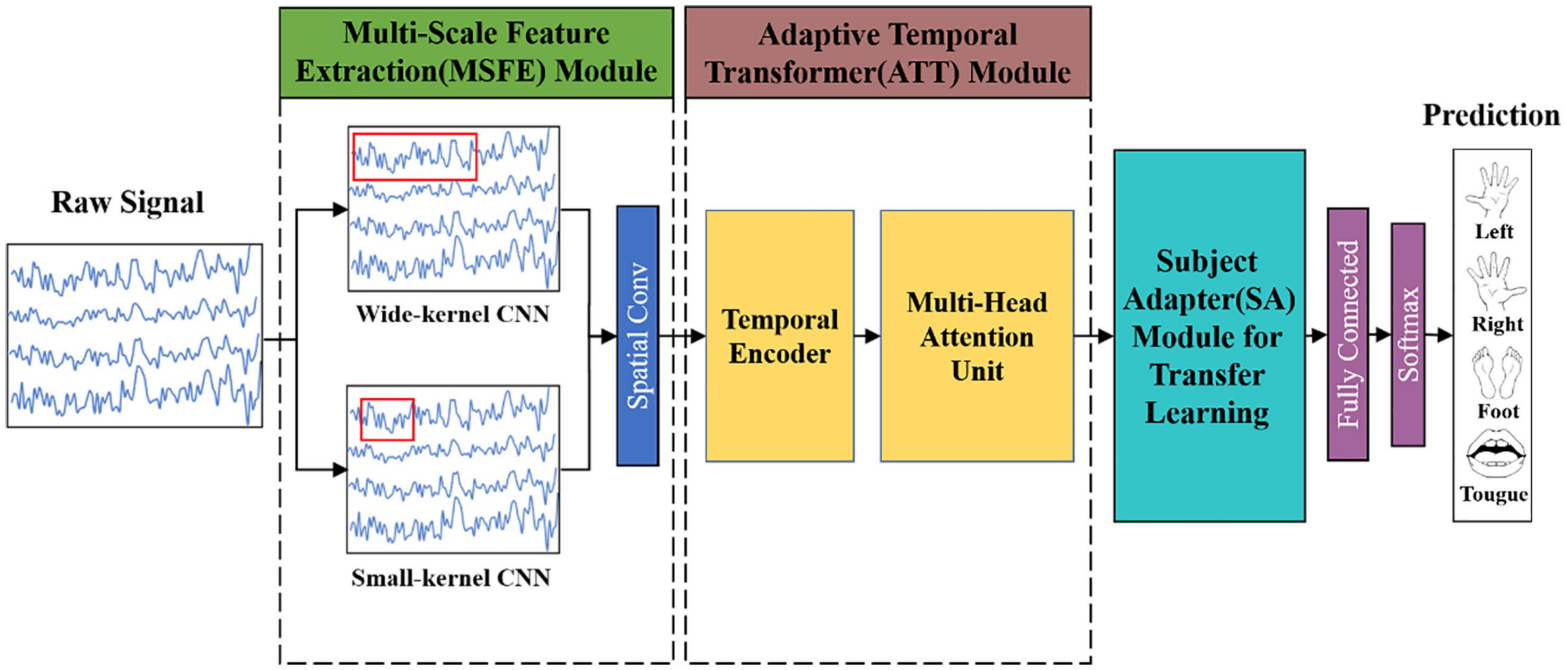
Diagram showing the general framework of the MSATNet. For the within-subject experiments, the model consists of the MSFE and ATT modules. For the cross-subject experiments, the model adds the SA module to the original model.

A multi-scale adaptive transformer model called MSATNet is proposed to decode the acquired EEG signals and obtain the user motor imagery awareness. The overall framework of MSATNet is shown in Figure 1. The MSATNet model consists of three modules: MSFE, ATT, and SA. First, a multiscale CNN network is used in the MSFE module to extract the input EEG signals from a local perspective. Large (small) convolutional kernels are used to extract the low (high)-frequency features. After the MSFE module, the ATT module adaptively extracts the temporal information of the EEG signal from a global perspective. This module consists of a temporal decoder and a multi-head attention unit. To enhance the cross-subject performance of the model, the SA module is introduced for fine-tuning the target subject data. Finally, the prediction category is output after a fully-connected layer and a softmax activation function.

The motor imagery recognition task can be defined as learning the nonlinear mapping between EEG signals and their corresponding categories:


$$Y_{class}=F\left(X\right)$$

Where X represents the representation of EEG signals, F denotes the learned nonlinear mapping, and $Y_{class}$ is the classification result.

### 2.4. Multi-scale feature extraction module

**Figure 2:**
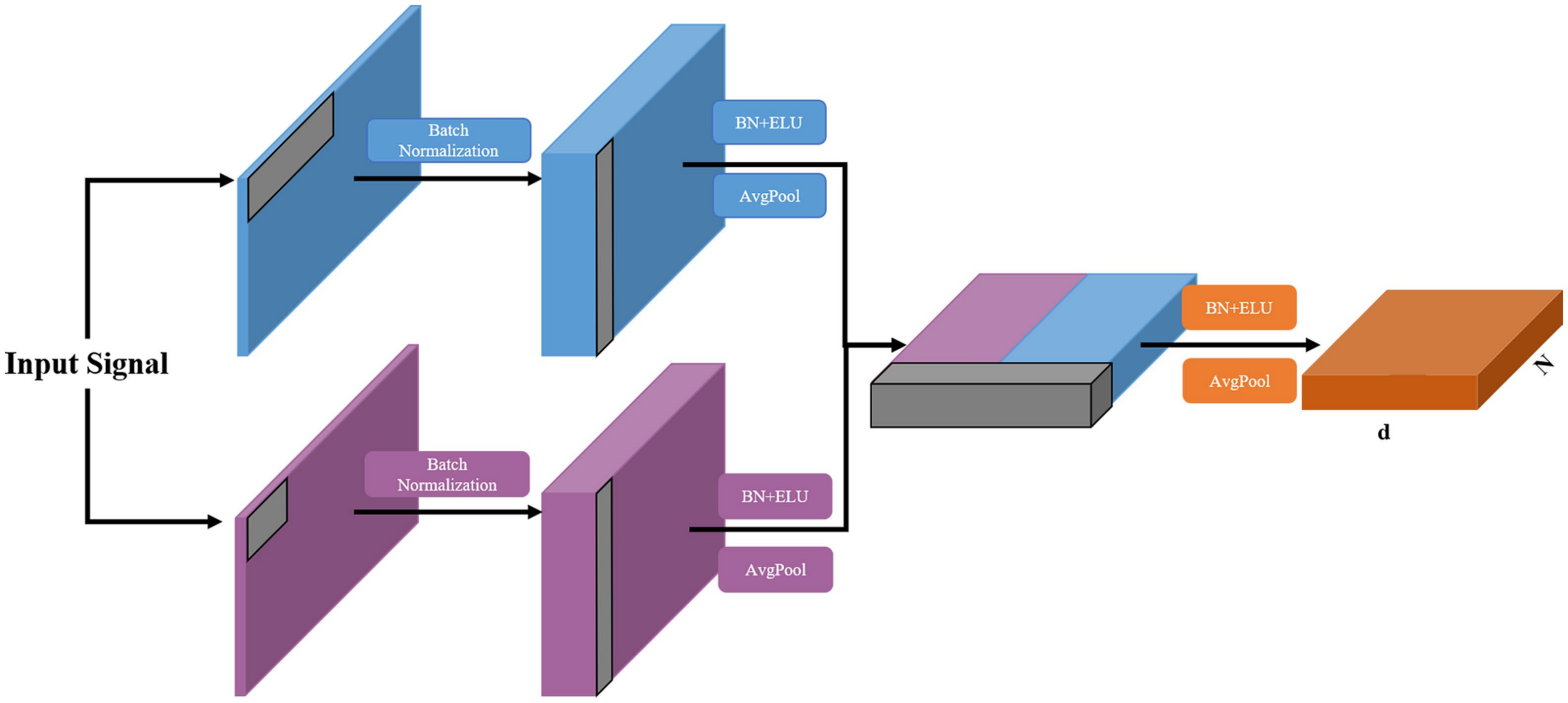
Structure of the proposed MSFE module, where N and d donate the sequence length and feature dimension, respectively.

The multi-branch convolutional structure can extract rich multi-scale signal features using variable-sized convolutional kernels in different branches, but too many branches make the model too parametric and prone to overfitting for small EEG datasets, which limits the enhancement of the classification performance. Therefore, we design an MSFE module with a two-branch structure, as shown in Figure 2. The MSFE is performed through two convolutional layers of different sizes, which ensures the richness of feature extraction while controlling the complexity of the model and avoiding overfitting. In each branch, the signals of each channel are first processed using one-dimensional temporal convolution as a frequency filter. Different convolution kernel sizes can capture various time step ranges and extract features from different frequency bands (Eldele et al., 2021). Therein, a large (small) convolution kernel is used to extract low (high)-frequency features. Then, depth-wise convolution is used to extract the features between the channels. After processing each branch with depthwise convolution, we fused the features and further processed the extracted multi-scale features with convolution. This approach improves upon previous methods, which utilized multiple branches and various convolutional layers, by reducing the number of parameters required for training while maintaining high classification accuracy. Each branch has two convolutional layers and an average pooling layer with each convolutional layer followed by batch normalization (Santurkar et al., 2018) and ELU function activation. The mathematical expression of the ELU function is:


$$ELU\left(x\right)=\{\begin{array}{cc} e^{x}-1 & x<0\\ x & x\geq 0 \end{array}$$

The fused features are then extracted in the feature dimension. To avoid overfitting, a dropout layer is added at the end of each branch and MSFE module. The detailed configuration of this module is provided in Table 1.

**Table 1:** Parameter settings for the proposed MSFE module, where p donates the dropout probability.

Layer	Filters	Size	Stride	Activation	Options
Conv2D	16	Branch1: (1,64) Branch2: (1,16)	1		
BatchNorm					
DepthwiseConv2D		(22,1)	1		
BatchNorm					
Activation				ELU	
AveragePooling2D		(1,8)	1		
Dropout					0.3
Conv2D	16	(1,16)	1		
BatchNorm					
Activation				ELU	
AveragePooling2D		(1,7)	1		
Dropout				ELU	0.3

### 2.5. Adaptive temporal transformer module

EEG signals contain rich temporal information. Previous methods often utilized TCN, GRU, and similar methods for feature extraction. However, these methods only capture information from a relatively small range, which is insufficient for EEG signals with long-term dependencies. Therefore, we designed an ATT module consisting of a temporal decoder and a multi-head self-attention mechanism unit. After the MSFE module, the temporal decoder extracts deeper temporal features, and the multi-headed attention unit focused on more important information in the time series. The temporal decoder is utilized to extract additional features and provide temporal encoding for the subsequent self-attention mechanism unit.

The TCN is distinct from the LSTM and GRU as it uses convolutions for sequence modeling, which can be processed in parallel and have a higher computational efficiency with lower memory requirements. We improve TCN and design a temporal decoder unit. After each original TCN, the ELU activation function is added to enhance the expressive ability of the model. The temporal encoder consists of a one-dimensional fully-convolutional network and a causal convolution. The one-dimensional fully-convolutional network uses zero-padding to ensure the input and output time steps are equal and that each time has a corresponding output. Causal convolution ensures that the features at each time point are determined only by the previous time points. A dilated convolution is introduced to expand the receptive field while avoiding layers that are too deep, which is the dilated casual convolution. For the input sequence $x\in {\mathbb{R}}^{n}$ , sequence element t , and filter f:{0,…,k−1}→ℝ , the dilated convolution F is calculated as:


$$F\left(t\right)=\left(x{*}_{d}f\right)\left(t\right)=\overset{k-1}{\underset{i=0}{\sum }}f\left(i\right)\;•\;x_{s-d\cdot i}$$

where d is the expansion factor, and k is the convolution kernel size.

**Figure 3:**
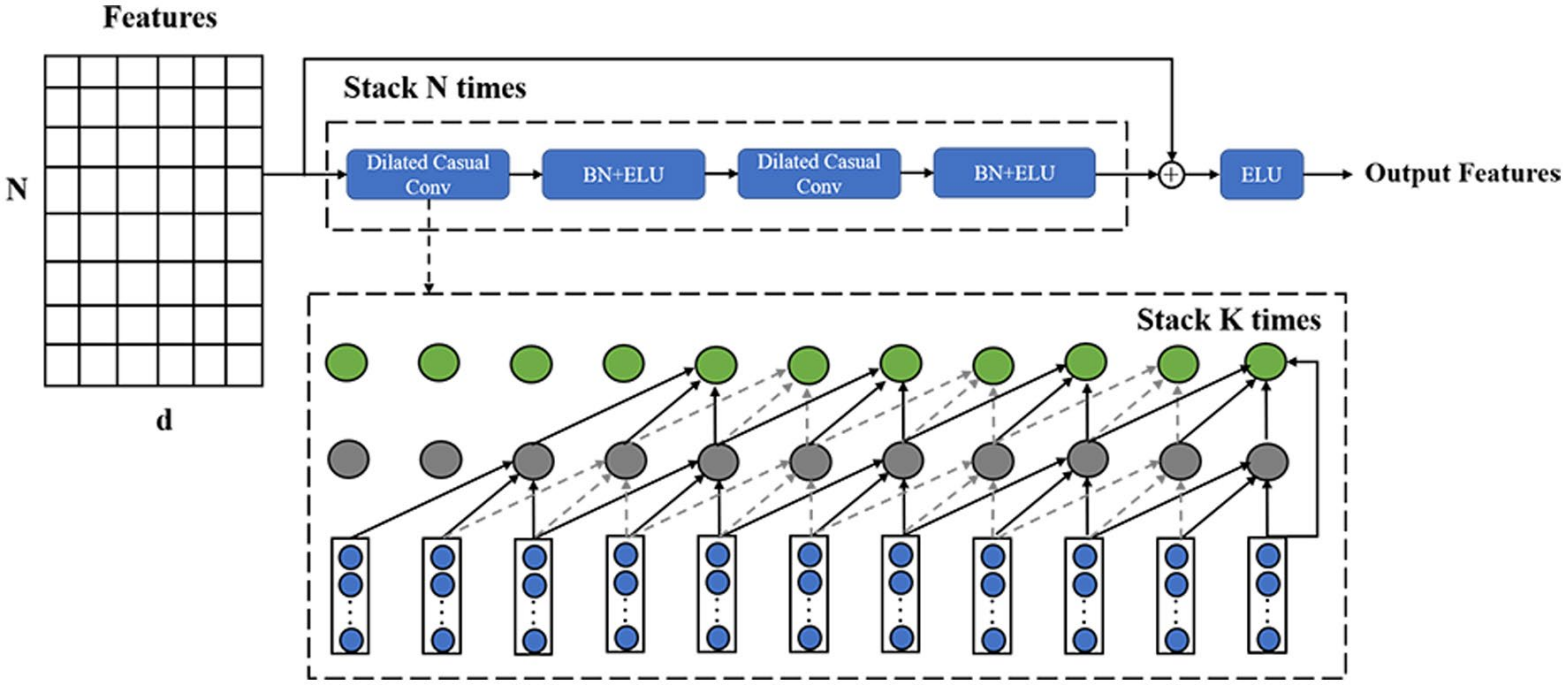
Structure of the temporal decoder, where the structure of the input features is the same as the previous module output.

The temporal decoder is implemented by the modified TCN, and the structure diagram is shown in Figure 3. We design the temporal decoder with two residual blocks to achieve a global perspective temporal feature extraction. Each residual block is composed of two layers of dilated casual convolutions, where the convolution expansion of the identical residual blocks is the same. The dilated casual convolution is accompanied by batch normalization and the ELU activation function after each convolution operation. For n residual blocks, m convolution layers in each block, convolution kernel of size K , and expansion of size b , the perceptual field of the temporal encoder is calculated as:


$$r=1+m\left(K-1\right)\frac{b^{n}-1}{b-1}$$

The attention mechanism imitates human cognitive attention, which enhances the weight of some parts of the input data while weakening the weight of others. This focuses attention in the network on the most important parts of the data. The self-attention mechanism is a variant of the attention mechanism, which reduces the dependence on external information and better captures the internal correlation of data or features. Vaswani et al. proposed the transformer model for sequence-to-sequence learning on text data and achieved a new state-of-the-art approach (Vaswani et al., 2017) that has been extended to various modern deep learning algorithms, including for language, vision, speech, and reinforcement learning. The transformer model is based entirely on the self-attention mechanism without any convolutional or recurrent neural network layers.

**Figure 4:**
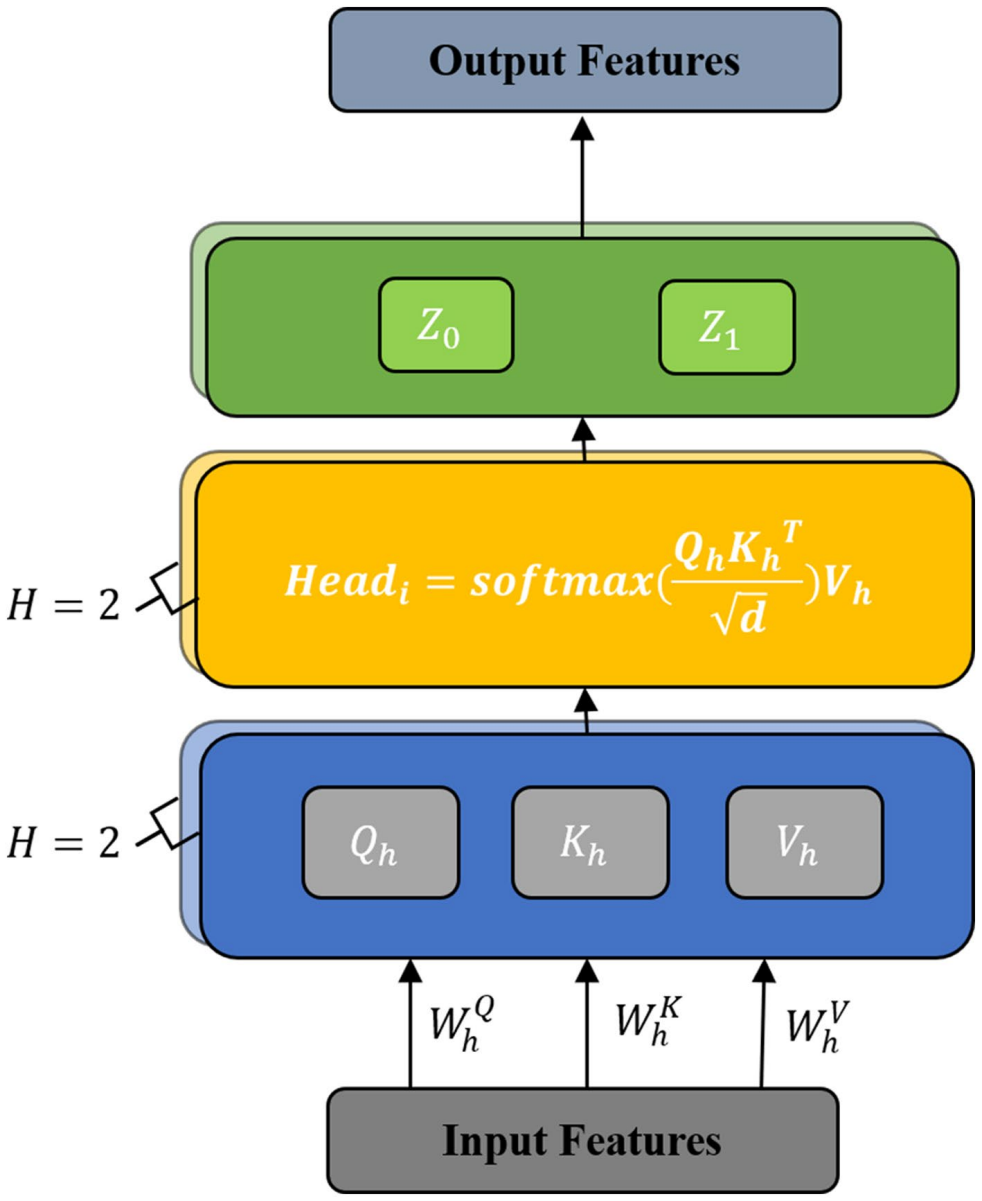
Structure of the multi-head attention unit.

We incorporated the design principles of transformers into motion recognition, but applying this method to EEG signals presents several challenges. Compared to the vast amount of language data used in transformer training, EEG data is limited, making it insufficient for training transformer architectures that require large amounts of data. Additionally, EEG signals are one-dimensional time-series data with high sampling frequencies, making it computationally intensive to directly input them into a transformer. To address these challenges, we utilized multi-scale feature extraction and temporal convolution to extract small-dimensional features that still contain rich signal information, which were then used as inputs for the multi-head self-attention unit. Furthermore, we reduced the model size by using only two parallel attention heads for computation, allowing us to maintain accuracy while reducing computational cost. Therefore, we designed a multi-head attention unit, which is mainly composed of a multi-head self-attention mechanism (Figure 4).

The multi-head attention mechanism sets multiple attention heads based on the self-attention mechanism, which divides the input features and increases the learning space of the model. This provides a mechanism to comprehensively focus on information at different times and representation subspaces, which enhances the model’s ability to decode complex information in EEGs.

The multi-head self-attention mechanism consists of multiple self-attention layers. Each self-attention layer is composed of a query Q , keys K , and values V . The features after the temporal decoder have the form $X\in R^{N\times d}$ , where N is the sequence length and d is the feature dimension. Under H heads, each X is split into H spaces, and the converted features are expressed as $X^{\prime }=\left\{X_{1},\ldots X_{H}\right\}$ , where $X\prime_{h}\in R^{N\times \frac{d}{H}}$ and 1 ≤ h ≤ H. Then, $Q_{h}$ , $K_{h}$ , and $V_{h}$ are multiplied by the transformation matrices $W_{h}^{Q}\in {\mathbb{R}}^{d\times d_{k}}$ , $W_{h}^{K}\in {\mathbb{R}}^{d\times d_{k}}$ , and $W_{h}^{V}\in {\mathbb{R}}^{d\times d_{v}}$ . The formulas are given as:


$$Q_{h}=W_{h}^{Q}X\prime_{h}$$

$$K_{h}=W_{h}^{K}X\prime_{h}$$

$$V_{h}=W_{h}^{V}X\prime_{h}$$

Next, we use the three matrices $Q_{h}$ , $K_{h}$ , and $V_{h}$ to calculate the attention score $\;Z_{h}$ of each attention head as:


$$Z_{h}=softmax\left(\frac{Q_{h}K_{h}{}^{T}}{\sqrt{d}}\right)V_{h}\in {\mathbb{R}}^{N\times \frac{d}{H}}$$

Finally, we connect the H representations and perform a spatial transformation to obtain the final output as:


$$MultiHead\left(Q,K,V\right)=Concat\left(Z_{1},\ldots ,Z_{h}\right)W^{o}\in {\mathbb{R}}^{N\times \frac{d}{H}}$$

where the projection is the parameter matrix $W^{o}\in {\mathbb{R}}^{Hd_{v}\times d}$ . For each of these, we use $d_{k}=d_{v}=d∕H$ .

### 2.6. Subject adapter module

**Figure 5:**
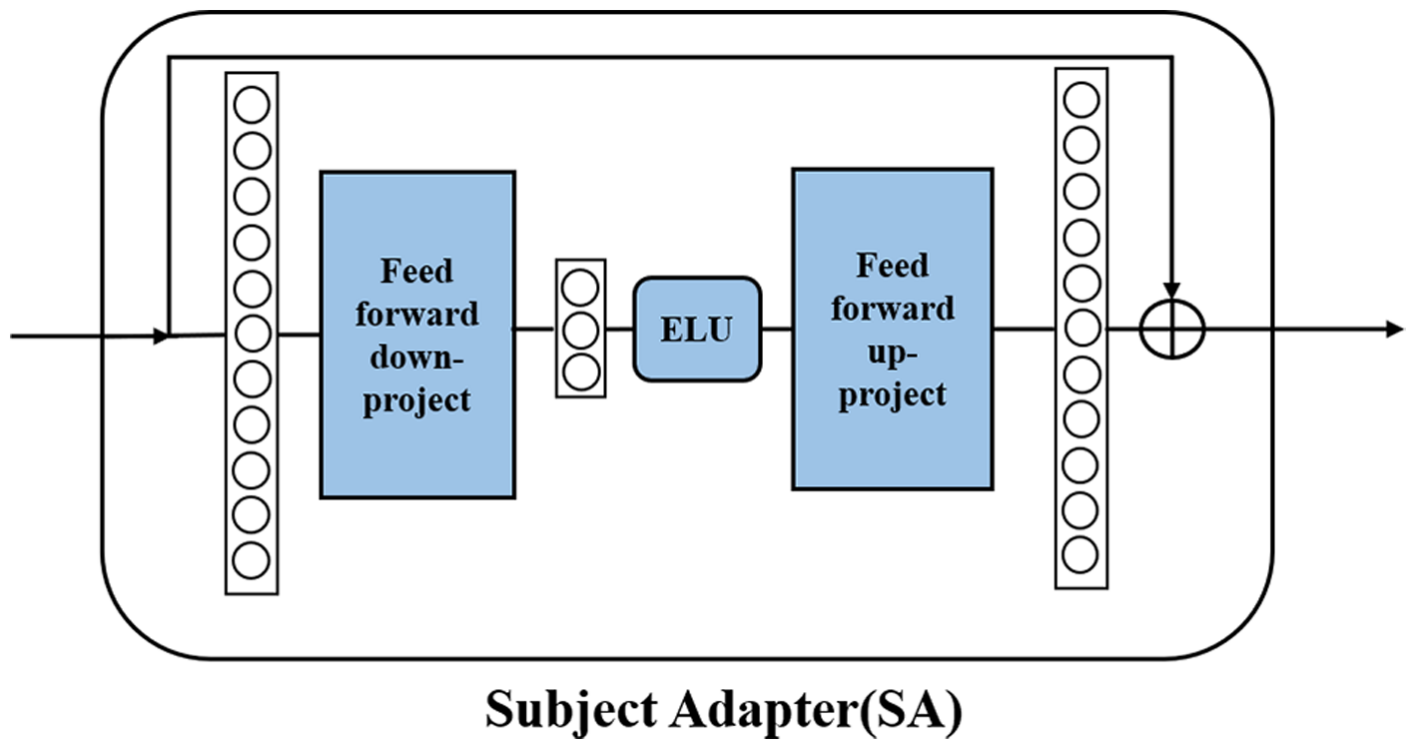
Structure of the SA module.

Due to the individual variability of EEG signals, how to improve the generalization ability of the model has always been a challenge. The original transfer learning method based on domain adaptation needs to obtain all the data of the new individual, which is obviously difficult to use in practice. To address the limitations of previous methods, we designed our own adapter module. By retraining the adapter module with partial data from new individuals, efficient transfer learning can be achieved. Detailed training and testing strategies will be described in the Experimental Setup section. As shown in Figure 5, the SA module is a bottleneck structure that consists of two feedforward layers and the ELU activation function. The feedforward down-project layer transforms high-dimensional features into low-dimensional features and then transforms them into their original dimension using the feedforward up-project layer after passing the ELU activation function. The SA module also contains a residual connection to prevent model performance degradation. The pre-trained fine-tuning approach obtains a more generalized model by pre-training on a larger source domain and then fine-tuning it using a smaller amount of data from the target domain. This method ensures a higher cross-subject performance with a shorter calibration time. The SA module can achieve subject-specific adaptation of the pre-trained model by introducing a very small number of parameters.

## 3. Experiments

This paper conducts within-subject and cross-subject experiments to illustrate the effectiveness of the MSATNet in classifying and identifying motor imagery signals. The within-subject experiments illustrate the effectiveness of MSATNet in EEG feature extraction, and the cross-subject experiments illustrate the effectiveness of MSATNet in model transfer after adding the SA module.

### 3.1. Benchmark models

We choose FBCSP, EEGNet, EEG-ITNet, and SHNN for within-subject performance comparison and DJDAN and JDAO-Mix for cross-subject performance comparison. The FBCSP applies the SVM classifier for classification by slicing bands and selecting features based on the CSP algorithm (Ang et al., 2008). The EEGNet applies frequency filters *via* temporal convolution, applies depth-wise convolution to learn frequency-specific spatial filters, and then applies separable convolution to learn the features from different feature maps (Lawhern et al., 2018). The EEG-ITNet performs multi-domain feature extraction using a multi-branch CNN and dilated casual convolution (Salami et al., 2022). The SHNN uses the SincNet-based CNN structure to extract spatial and spectral features of EEG signals, uses the SE module to recalibrate the features to obtain a sparse representation of the EEG, and applies the GRU module to extract the sequence relationship of the data (Liu et al., 2022). The DJDAN uses temporal and spatial convolutions for feature extraction and applies an adversarial learning strategy for domain adaptation (Hong et al., 2021). The JDAO-Mix uses optimal transport for joint distribution adaptation, which is the latest SOTA method (Chen et al., 2022).

### 3.2. Experimental setup

For the within-subject experiments, only the MSFE and ATT modules were available as the SA module was not added. Both the training and test data were obtained from the same subject. The training set was taken from session 1 and the test set was from session 2; thus, the effect of different sessions on the EEG data was ignored. We used the training set from each subject for training and then performed validation with the test set from the corresponding subject. The EEG data were taken from the dataset without any other pre-processing. The data division standard for all comparison methods is the same to ensure a fair comparison. The EEG-ITNet is tested under the same conditions as the proposed model, and the results of the remaining comparison models are taken from their original papers. We apply accuracy as the evaluation metric.

For the cross-subject experiments, the SA module is added, and the model evaluation is divided into four parts. (1) We first divide the entire dataset into two parts: the i -th subject is a randomly-selected target domain, and the remaining (N−1) subjects are the source domain. Half of the samples in the target domain are used for fine-tuning, while the other half is used to evaluate the classification performance. (2) We pre-train the proposed model on all samples of the source domain and update all trainable parameters. (3) Based on the training samples of the target domain, we only fine-tune the parameters embedded in the SA module to narrow the gap between the target and source domains. (4) Finally, the trained model is evaluated on the test samples of the target domain. The EEG data were taken from the dataset without any other pre-processing. The results of the benchmark models were taken from their original papers. The model parameters, evaluation indexes, and experimental assumptions were the same as those of the within-subject experiments.

We built the model in TensorFlow and trained it using an RTX 3060 GPU. The batch size was set to 64 and the Adam optimizer was used with a learning rate of 0.0008. All convolutional layers were initialized using parameters based on the Glorot method, and the final fully-connected layer was constrained with maximum parametric weights having a parameter value of 0.25. The default epoch of the experiments was 1,000 iterations, and an early stopping mechanism was used to prevent overfitting. For the temporal decoder, the convolution kernel size was 4 and the expansion factor was 2. We used 2 attention heads in parallel for the multi-head attention unit.

## 4. Results and analysis

This section analyzes the experimental results of MSATNet in the within-subject experiments for the BCI Competition IV 2a and 2b datasets and describes the effects of the MSFE and ATT modules with the ablation experiments in the BCI Competition IV 2a dataset. Then, the experimental results of MSATNet in the cross-subject experiments in the BCI Competition IV 2a and 2b datasets are analyzed, and the effects of the SA module are considered in the ablation experiments from the BCI Competition IV 2a dataset.

### 4.1. Within-subject experimental results and analysis

#### 4.1.1. Analysis of model effect

**Figure 6:**
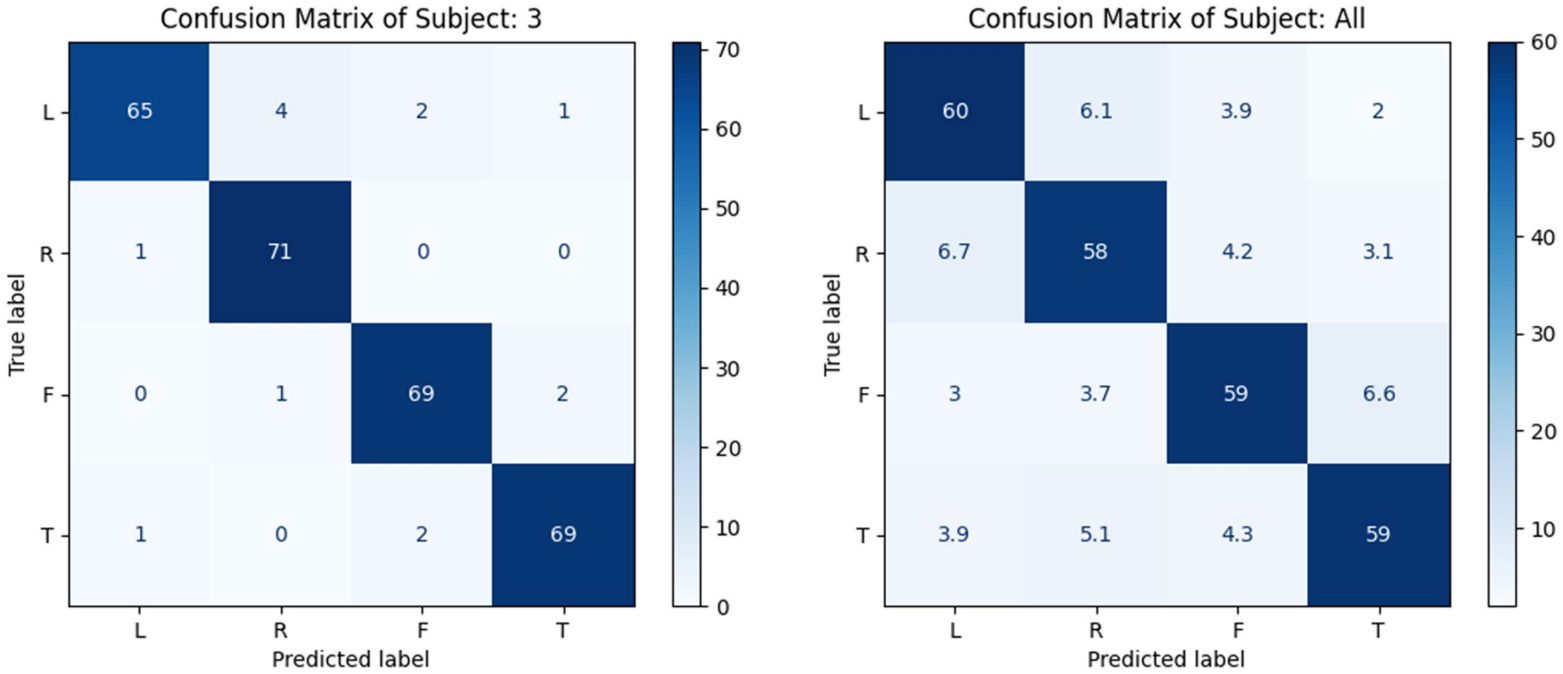
Confusion matrix of a single subject (left, S3 as a representative) and of all subjects (right, with mean value processing), where L, H, F, and T are abbreviations for left-hand, right-hand, feet, and tongue, respectively.

To evaluate the performance of the model for the within-subject experiments, the proposed MSATNet model was tested on the BCI Competition 2a and 2b datasets, and the experimental results were compared with FBCSP (Ang et al., 2008), EEGNet (Lawhern et al., 2018), EEG-ITNet (Salami et al., 2022), and SHNN (Liu et al., 2022) models. Table 2 summarizes the accuracy of the different subjects and average accuracy under the BCI Competition IV 2a dataset for the proposed and benchmark methods. The powerful feature extraction ability of the neural network gives a higher classification performance for deep learning methods than in traditional machine learning approaches. In particular, the proposed MSATNet achieved the greatest accuracy among most subjects and the highest average accuracy. Compared with EEGNet, we use multi-scale convolution and adaptively extract features from a global perspective, which achieves a performance improvement of 8.06%. Compared with EEG-ITNet and SHNN, we use the multi-head attention unit to focus on information related to EEGs and attain a higher accuracy in EEG recognition. Figure 6 shows the confusion matrix of the number of correct and incorrect predictions generated under the BCI competition IV 2a dataset. As can be seen in Figure 6, the data in the confusion matrix is mainly distributed on the diagonal of the matrix, indicating that MSATNet correctly predicted most of the imagined actions of the subjects with a low error rate.

**Table 2:** Classification performance of the MSATNet and benchmark models using the BCI Competition IV 2a dataset for the within-subject experiments.

Subject	FBCSP	EEGNet	EEG-ITNet	SHNN	Proposed method
S1	76.00	81.94	84.38	82.76	**90.62**
S2	56.50	56.95	62.85	**68.97**	65.97
S3	81.25	90.62	89.93	79.31	**95.14**
S4	61.00	67.01	69.1	65.52	**78.12**
S5	55.00	72.57	74.31	58.62	**79.86**
S6	42.25	58.68	57.64	48.28	**62.50**
S7	82.75	76.04	88.54	86.21	**91.67**
S8	81.25	81.25	83.68	**89.66**	88.89
S9	70.75	78.12	80.21	**89.87**	82.99
Mean	67.42	73.69	76.76	74.26	**81.75**

To further verify the effectiveness of the proposed model, it was tested on the BCI Competition IV 2b dataset. Table 3 shows the accuracy of different subjects and average accuracy under the BCI Competition IV 2b dataset based on the proposed and benchmark methods. The proposed model still achieves the highest accuracy in most subjects and achieves the highest average accuracy. The data show that the performance of the FBCSP is greater than that of the EEGNet, but the FBCSP has the worst performance in the BCI Competition IV 2a dataset. This indicates that the generalization performance of the method extracted by the artificial design is poor, and the extracted features cannot be widely applied. However, the remaining deep learning methods perform better in both datasets. The MSATNet achieves effective feature extraction through the MSFE module extracting multi-band rich features and the ATT module adaptively capturing the temporal dependencies of EEG signals. In summary, the MSATNet performs well on both datasets and surpasses the benchmark methods in terms of performance. Thus, MSATNet can identify users’ motor imagery consciousness more accurately when used for motor imagery BCI data extraction.

**Table 3:** Classification performance of the MSATNet and comparison benchmark methods using the BCI Competition IV 2b dataset for the within-subject experiment.

Subject	FBCSP	EEGNet	EEG-ITNet	SHNN	Proposed method
S1	70.00	67.50	67.5	**83.33**	81.87
S2	60.36	60.35	71.43	61.76	**72.14**
S3	60.94	62.81	86.88	58.33	**87.81**
S4	97.50	91.25	**98.44**	97.30	97.81
S5	93.12	83.44	94.06	91.89	**99.38**
S6	80.63	61.56	86.25	**88.89**	88.44
S7	78.13	83.75	90.00	86.11	**93.13**
S8	92.50	91.88	93.44	92.11	**94.37**
S9	86.88	82.50	55.31	**91.67**	89.06
Mean	80.00	76.12	82.59	83.49	**89.34**

#### 4.1.2. Analysis of the effect of the MSFE module

**Figure 7:**
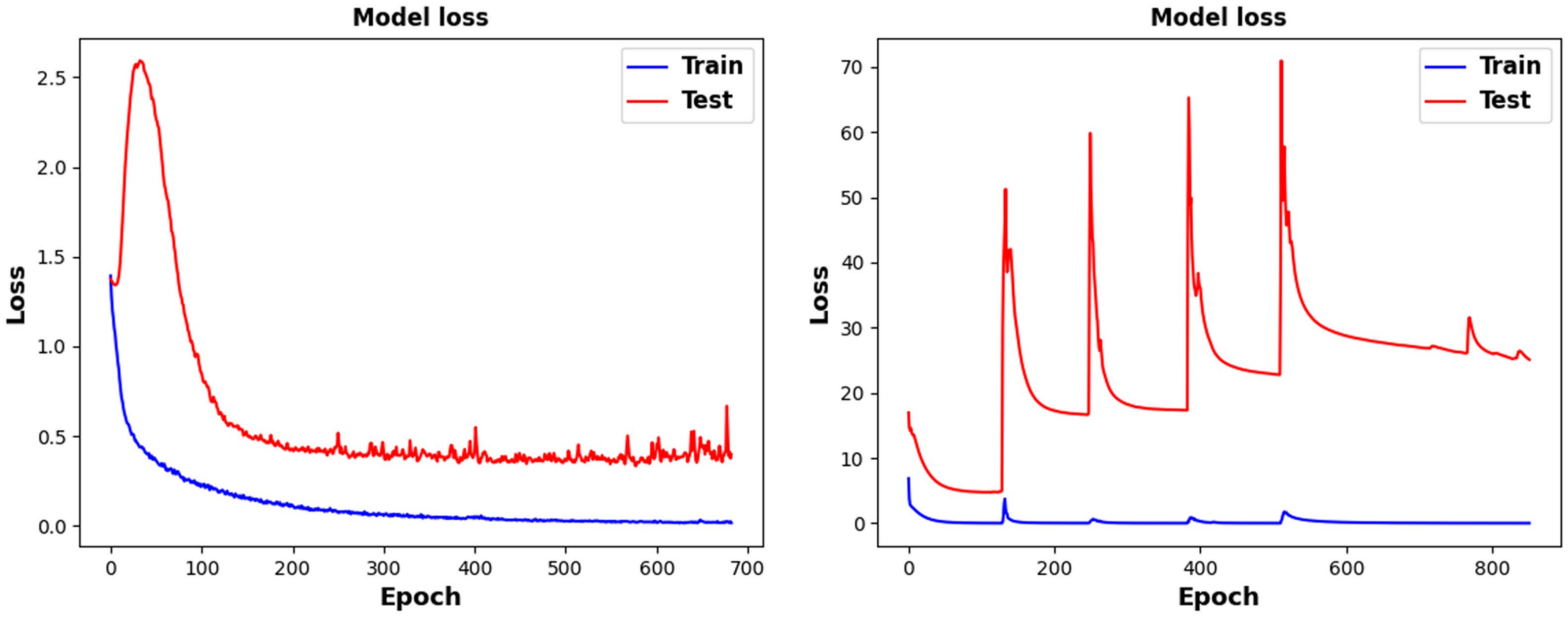
Classification performance of different branches for the MSFE module.

Five models were used for comparative experiments to explore the impact of the MSFE module on the classification accuracy. (1) A single-scale feature extraction model that only retains a single branch with a convolution kernel size of 64. (2) The MSATNet model, which has the 2-branch structure shown in Figure 2. (3) A three-branch feature extraction model with three convolution kernels sized at 64, 32, and 16. (4) Three convolution kernels with sizes of 64, 32, 16, 8, and 4 are used with a five-branch feature extraction model. (5) The existing multi-branch multi-scale model MMCNN (Jia et al., 2021), which replaces the MSFE module in MSATNet while keeping the rest of the model the same. We conducted within-subject classification experiments using these five models on the BCI Competition IV 2a dataset. The accuracy of different subjects and the average accuracy are shown in Figure 7.

**Figure 8:**
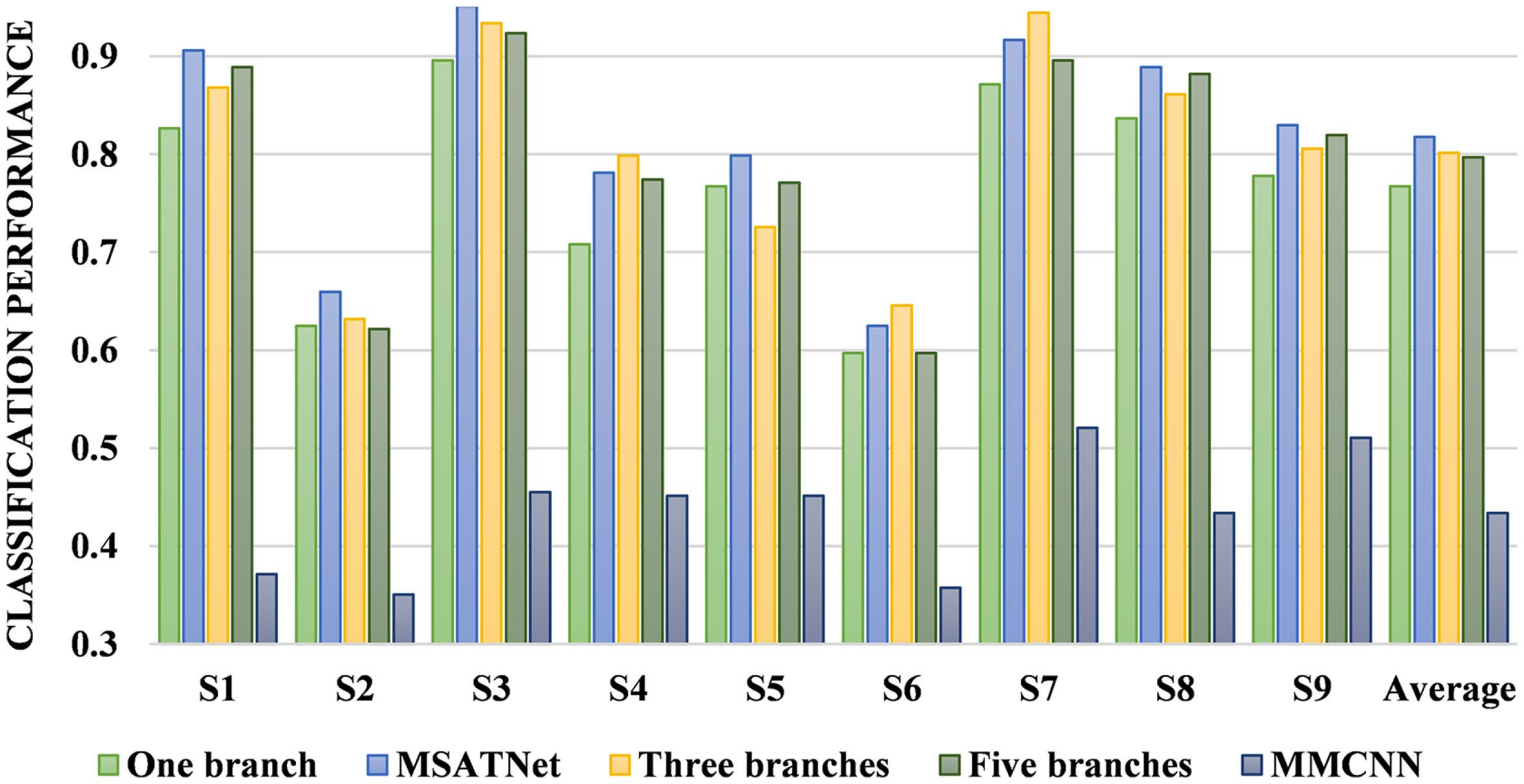
Model loss change diagram during training and testing. The left picture is the MSATNet, and the right picture is the comparison model MMCNN.

The two-branch feature extraction model can extract more abundant signal features than the single-branch model, but by adding more branches, feature redundancy may occur when using a three- or five-branch feature model, which degrades the classification performance. This is why the final design adopts two branches. Figure 8 shows that the complexity of the comparison model gives a significant overfitting phenomenon when faced with EEG datasets having a small amount of data. This significantly reduces the classification effect. At the same time, the MMCNN has a poor performance compared with the comparison model with five branches, indicating that the convolutional structure can effectively extract features. Additionally, the number of parameters (5.7 $\times 10^{4}$ ) of the multi-scale multi-branch module in MMCNN is approximately twice that of the proposed multi-scale feature extraction module (3.1 $\times 10^{4}$ ). Under the same experimental conditions, the training time of the MSATNet is about 32.7% that of the MMCNN. Therefore, the MSFE module can ensure a moderate complexity and extract more abundant EEG features.

#### 4.1.3. Analysis of the effect of the ATT module

**Figure 9:**
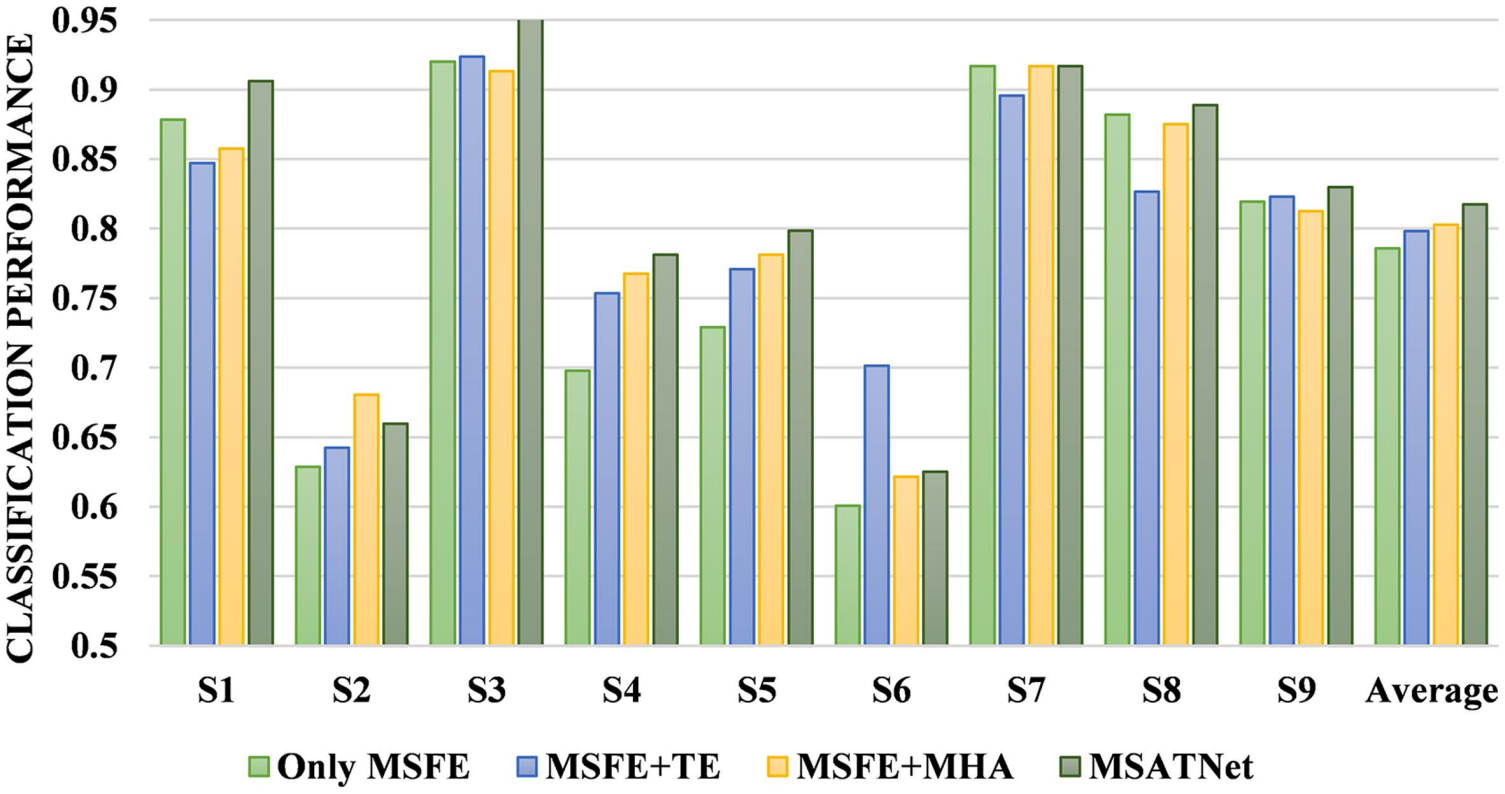
Classification performance of the ATT module ablation experiment.

We designed an ablation experiment to verify the effectiveness of the ATT module at improving the accuracy of EEG recognition. Four models are used for the comparative experiments. The first comparison model is composed of only the MSFE module, the second is composed of the MSFE module and temporal decoder, the third is composed of the MSFE module and multi-head attention unit, and the fourth is the MSATNet. Figure 9 shows the accuracy of the different subjects and the average accuracy of the four comparison models under the BCI Competition IV 2a dataset. The model performance improved after adding the temporal decoder due to the feature extraction of the local temporal information. Feature extraction of the multi-head attention mechanism improved the classification performance after adding the attention mechanism. However, the classification performances of the model adding only the temporal decoder or only the multi-head attention unit are lower than MSATNet. It shows that on the basis of local feature extraction, the ATT module further adaptively extracts shallow features from a global perspective. The experimental results show that the ATT module can effectively capture information related to motor imagery and improve the recognition accuracy.

### 4.2. Cross-subject experimental results and analysis

#### 4.2.1. Analysis of model effect

To evaluate the cross-subject classification performance of the model, we compare MSATNet with the benchmark models DJDAN (Hong et al., 2021) and JDAO-Mix (Chen et al., 2022) on the BCI Competition 2a and 2b datasets. In addition to performing well on the within-subject experiments, our method has good performance on the cross-subject experiments. We use eight subjects in the dataset for pre-training and the remaining subject for fine-tuning and performance evaluation. Table 4 shows the accuracy of different subjects and the average accuracy of the proposed and benchmark methods under the BCI Competition IV 2a and 2b datasets, respectively. The cross-subject classification accuracies of the model reached 81.33 and 86.23% respectively, which exceeds the DJDAN and JDAO-Mix models. After the MSFE module and ATT module, the model learns features with high discrimination and achieves efficient transfer in target subjects using only a small number of parameters. This method does not need to obtain all the target subject data, which shortens the tedious calibration time of the EEG and is important in practical applications.

**Table 4:** Classification performance of the MSATNet with benchmark methods using the BCI Competition IV 2a and 2b datasets in cross-subject experiments.

Method	BCI IV 2a	BCI IV 2b
DJDAN	53.20	76.24
JDAO-Mix	60.69	76.65
Proposed MSATNet	**81.33**	**86.23**

#### 4.2.2. Analysis of the effect of the SA module

**Figure 10:**
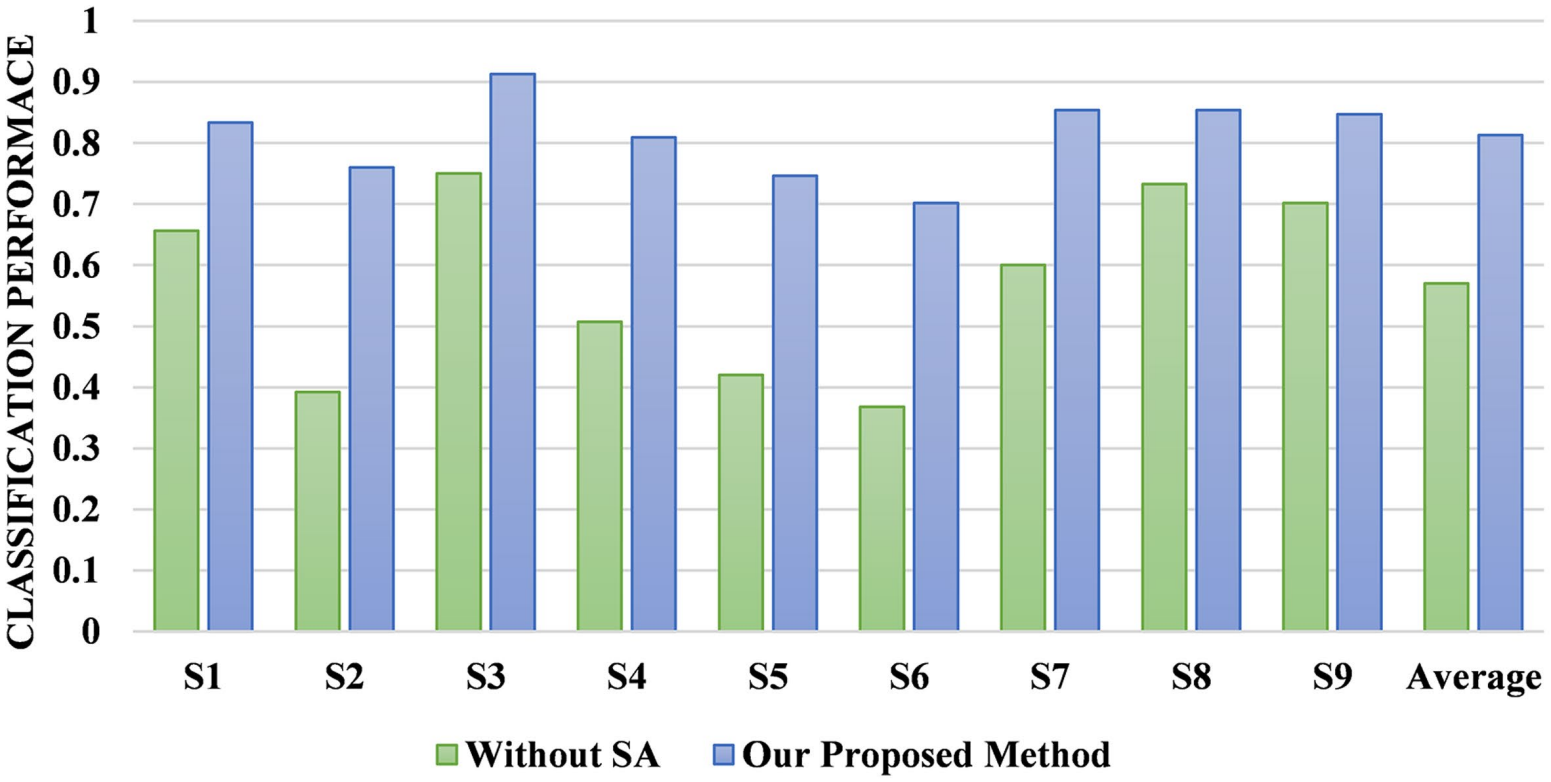
Comparison of the MSATNet and MSATNet with the SA module removed.

We designed two comparison models to verify the effectiveness of the SA module at improving the cross-subject performance. One model is composed only of the MSFE module and the ATT module and is directly tested on the target subjects after pre-training on eight subjects without the SA module for fine-tuning. The other model is composed of the MSFE, ATT, and SA modules, and the target subjects is adapted by fine-tuning the SA module. Figure 10 shows the accuracy of the different subjects and average accuracy under the BCI Competition IV 2a dataset for the model with and without the SA module. The MSATNet performs poorly in the face of new subjects, and features learned by the MSFE and ATT modules do not have better generalization. The addition of the SA module allows complete adaptation of the target domain with only a very small increase in the number of parameters to obtain better cross-subject performance under the common feature distribution of the learned source domain. Thus, the SA module helps adapt the model to the target subjects and can achieve more accurate transfer learning.

## 5. Conclusion

We propose a multi-scale adaptive transformer network called MSATNet. First, the MSFE module extracts rich features in different frequency bands. Then, the ATT module adaptively learns the information related to motion imagery from a global perspective. Finally, we achieve effective transfer learning with relatively few extra parameters using the SA module. Our approach was evaluated on two publicly available datasets, and the results indicate that it outperforms existing methods. Future work will extend the methodology to additional activities to include disease diagnostics. Although our work achieves good classification performance, it still has some limitations. First, we only recognize motor imagery patterns of different limbs, such as the left hand, right hand, and foot. In the future, our proposed model will distinguish more complex motor imagery patterns, such as small arm rotation and elbow flexion (Chu et al., 2020), which is important for patients’ rehabilitation training. Second, our adapter-based approach still requires data from the user for calibration, but the calibration process is time-consuming and requires significant time. Therefore, future work will push toward calibration-free BCI classification techniques.

## Data availability statement

Publicly available datasets were analyzed in this study. The datasets for this study can be found at: http://www.bbci.de/competition/iv/.

## Author contributions

LH conceptualized the study, presented the main idea of this manuscript. WH performed the experiments and analyses, wrote the first draft of the manuscript. All authors contributed to the article and approved the submitted version.

## Funding

This work was partially supported by the National Natural Science Foundation of China under grants 81960327, the foundation of Science and Technology Department of Shanghai of China under grant 23010501700, and the foundation of Health Commission of Jiangxi Province under (Grant NO2023ZD008).

## Conflict of interest

The authors declare that the research was conducted in the absence of any commercial or financial relationships that could be construed as a potential conflict of interest.

## Publisher’s note

All claims expressed in this article are solely those of the authors and do not necessarily represent those of their affiliated organizations, or those of the publisher, the editors and the reviewers. Any product that may be evaluated in this article, or claim that may be made by its manufacturer, is not guaranteed or endorsed by the publisher.
